# A redescription of the leggiest animal, the millipede *Illacme plenipes*, with notes on its natural history and biogeography (Diplopoda, Siphonophorida, Siphonorhinidae)

**DOI:** 10.3897/zookeys.241.3831

**Published:** 2012-11-14

**Authors:** Paul E. Marek, William A. Shear, Jason E. Bond

**Affiliations:** 1 University of Arizona, Department of Entomology, Forbes Building, Tucson, Arizona, USA; 2 Hampden-Sydney College, Department of Biology, Gilmer Hall, Hampden-Sydney, Virginia, USA; 3 Auburn University, Department of Biological Sciences, Funchess Hall, Auburn, Alabama, USA

**Keywords:** California Floristic Province, paleoendemic, endemic, silk, San Benito County, Silicon Valley, Salinas Valley, sandstone, burrowing, conservation, Gabilan Range

## Abstract

With up to 750 legs, the millipede *Illacme plenipes* Cook and Loomis, 1928 is the leggiest animal known on Earth. It is endemic to the northwestern foothills of the Gabilan Range in San Benito County, California, where it is the only known species of the family Siphonorhinidae in the Western Hemisphere. *Illacme plenipes* is only known from 3 localities in a 4.5 km^2^ area; the 1926 holotype locality is uncertain. Individuals of the species are strictly associated with large arkose sandstone boulders, and are extremely rare, with only 17 specimens known to exist in natural history collections. In contrast with its small size and unassuming outward appearance, the microanatomy of the species is strikingly complex. Here we provide a detailed redescription of the species, natural history notes, DNA barcodes for *Illacme plenipes* and similar-looking species, and a predictive occurrence map of the species inferred using niche based distribution modeling. Based on functional morphology of related species, the extreme number of legs is hypothesized to be associated with a life spent burrowing deep underground, and clinging to the surface of sandstone boulders.

## Introduction

The millipede *Illacme plenipes* has more legs than any other known organism, with one female individual possessing 750 legs on 192 body segments. The Siphonophorida, the order in which *Illacme plenipes* is placed, comprises a diversity of taxa that have fascinating anatomical features, biogeographical patterns, and very intriguing biology. Siphonophoridan species are mainly Pantropical in distribution with a few outlying taxa in the Himalayas, New Zealand, South Africa and California (Shelley and Golovatch 2011). Despite their interesting biological and life history characteristics and a relictual distribution pattern, the group has been deemed a “taxonomist’s nightmare” and is among the least popular taxa in Diplopoda (Hoffman 1980; Jeekel 2001; Read and Enghoff 2009). At present, two families are recognized in the order: Siphonophoridae and Siphonorhinidae. Among these families, there are three genera of Siphonophorida in the United States: *Siphonophora*, *Siphonacme* and *Illacme*. The first two are classified as Siphonophoridae, while *Illacme* is the only known Western Hemisphere representative of Siphonorhinidae.

Like many other colobognath millipedes, the Siphonophorida often occur in cryptic subterranean habitats, shun light, are infrequently encountered, and therefore are rare in natural history collections. All known taxa are eyeless and have relatively large antennae. Species of the family Siphonophoridae have the front of the head drawn out into a long, narrow extension that is paralleled by a similar extension of the gnathochilarium, forming a tube that encloses reduced, stylet-like mandibles. Fungivory, the consumption of soft fungal tissues and spores, may be linked to this suite of adaptations. Siphonorhinids, in contrast, do not have this “beak” and the head is not strongly modified. The siphonorhinid gnathochilarium has all of its elements indistinguishably fused and is tightly appressed to the ventral surface of the head, leaving only a small opening anteriorly, which may be homologous to the labral indentation in eugnathan millipedes.

The cuticle of *Illacme plenipes* isadorned with a surprising diversity of peculiarly shaped spines, teeth, setae, sensilla, and other phaneres. Numerous setae clothing the dorsum of the millipede appear to secrete a viscous silk-like substance. The posterior one-third of its gut (the metenteron) is spiraled and visible through its translucent exoskeleton.

*Illacme plenipes* was described by O.F. Cook and H.F. Loomis in 1928 from seven individuals collected from a site located “a short distance after crossing the divide between Salinas and San Juan Bautista…in a small valley of a northern slope wooded with oaks, under a rather large stone” (Cook and Loomis 1928: 12). Cook and Loomis described the species (and genus) without an illustration or image and provided a short differential diagnosis distinguishing it from the other U.S. Siphonophorida species, *Siphonophora* and *Siphonacme*. Based on specimens examined from the type series, Shelley (1996b) provided the first illustrations of the genus and species, and reviewed the current knowledge of the order Siphonophorida in North America some seventy years later. To our knowledge, the species was not seen again in the wild for almost 80 years.

In 2005 and 2007, new specimens were collected from near the type locality (Marek and Bond 2006), as described below. The rediscovery of the species was detailed by Marek and Bond (2006) and included first-ever live video of the species, natural history observations and scanning electron micrographs of the external anatomy. These recent specimens, and previously collected material conserved in various museums, form the basis of the detailed redescription provided here.

## Fieldwork

Following the locality description of Cook and Loomis (1928), oak valleys in San Benito and Monterey counties were searched for populations of *Illacme plenipes* by P.E.M. and J.E.B in 2005. We focused collecting beside roads connecting the cities of Salinas and San Juan Bautista in the northwestern half of the Gabilan Range, from Fremont Peak northwest to Pinecate Peak and U.S. Highway 101. We thoroughly covered areas on the north slopes of the Gabilan Range closer to San Juan Bautista because the type locality specifically mentions the city, and moister conditions exist on the north-facing slopes. We also (in 2006) searched nine localities in a 67.5 km radius around the site where we rediscovered populations of *Illacme plenipes* in 2005. We visited the following localities: Frank Raines Park, Henry Coe State Park, Fremont Peak State Park, Pinnacles National Monument, Mount Madonna County Park, Alum Rock, Joseph D. Grant County Park, El Rancho Cienega del Gabilan and a private ranch near San Juan Bautista. Google Maps (Mountainview, CA), USGS geological maps, and topomaps in ACME Mapper 2.0 (Acme Labs, Berkeley, CA) were examined for suitable localities to search for populations of *Illacme plenipes*. These localities were chosen prior to estimating *Illacme plenipes*’ ecological niche (see methods below). We focused on valleys and oak woodlands because they too are moister. The underside of decaying oak logs and stones were examined for millipedes. When an individual was encountered, featherweight forceps were used to gently lift the millipede and place it into a collecting vial. Geographical coordinates were recorded, and significant biotic and abiotic features were documented. Specimens were each given unique numbers and maintained alive in collecting vials for between 2 – 10 days to photograph, record video footage and observe behavior and locomotion.

### Ecological niche modeling

As an approach to understanding species ecology and geography, a niche-based distribution model (DM) was constructed for *Illacme plenipes*. Niche-based DMs provide estimates for the probability of finding a species at a particular location and general area on a landscape given a known set of coincident ecological and climatic parameters for the species. Locality coordinates for each species were imported into ArcMap (ESRI, Redlands, CA) and converted into shape files. Following the procedure outlined in Bond and Stockman (2008) and Walker et al. (2009), DMs were constructed using environmental layers thought to “likely influence the suitability of the environment” (Phillips et al. 2006) based on previous analyses of other California-distributed taxa (see Stockman and Bond 2007, for further justification of layer choice). Seven climatic layers were obtained from the WORLDCLIM data set (Hijmans et al. 2005): annual precipitation, precipitation seasonality, annual maximum temperature, annual minimum temperature, temperature seasonality, and mean precipitation during the driest and wettest quarters. A seventh layer, elevation, was constructed from a mosaic of Digital Elevation Models (DEMs) derived from the National Elevation Dataset (USGS). DEMs were converted to raster format in ArcMap and resampled from 30-m resolution to 1-km resolution using bilinear interpolation. All seven layers were clipped to the same extent, cell size, and projection. Niche-based DMs were created using the computer program Maxent (Phillips et al. 2006). Maxent employs a maximum likelihood method that estimates a species’ distribution with maximum entropy subject to the constraint that the environmental variables for the predicted distribution must match the empirical average (Elith et al. 2006; Phillips et al. 2006). Parameters for all Maxent analyses used the default values: convergence threshold = 10−5, maximum iterations = 500, regularization multiplier = 1, and auto features selected. Additional larger values of the regularization multiplier were used to ensure that models were not overfitting the data.

### Specimen preservation

Specimens from which DNA was not extracted (typically longer females possessing more than 170 segments) were directly preserved in 80% ethanol. The posterior seven segments of two specimens (# SPC000924 and SPC001187) were dissected from live individuals with flame-sterilized forceps and stored in RNAlater (Qiagen Inc., Valencia, CA) at 10°C for 24h, and subsequently at -80°C for long-term preservation and archival storage of DNA and RNA. The enteron was removed from the segments to prevent contamination due to the DNA or RNA of the millipede’s gut contents. Specimens from which DNA was extracted were subsequently preserved in 70% isopropanol.

### DNA barcoding

Genomic DNA was extracted from frozen tissue preserved in RNAlater using standard DNeasy tissue extraction protocol (Qiagen Inc., California). Extracted DNA was purified from a fragment of the millipede (specimen #SPC001187) approximately four segments in length, with remaining tissue archived at -80°C in RNAlater. Genomic DNA is archived in Qiagen AE buffer at -20°C and stored in the cryo-collections at the University of Arizona and Auburn University. A region of DNA from the cytochrome c oxidase I gene (COI), was amplified using polymerase chain reaction (PCR) with the universal DNA barcoding primers of Folmer et al. (1994): LCO1490 (5’-GGT CAA CAA ATC ATA AAG ATA TTGG-3’) and HCO2198 (5’-TAA ACT TCA GGG TGA CCA AAA AAT CA-3’). This region corresponds to the *Drosophila* COI region: 1057 – 1500. PCR amplifications were cleaned, quantified and sequenced at Auburn University (AU Genomics and Sequencing Laboratory, Auburn, AL) on an ABI 3100 capillary DNA sequencer. For diagnostic identification purposes, COI barcoding DNA from commonly encountered colobognathan millipedes that co-occur with *Illacme plenipes* in the western U.S. (*Gosodesmus claremontus*, *Brachycybe producta*, *Brachycybe rosea*, and *Siphonacme lyttoni*) and may be confused with the species, was extracted, amplified and sequenced in an identical manner to provide a database of sequences against which unknown query sequences can be compared. Sense and antisense COI sequence chromatograms were processed using Phred and Phrap in the Mesquite ver. 2.75 module Chromaseq (ver. 1.0), which includes matching contiguous regions and base call quality scoring (Ewing and Green 1998; Maddison and Maddison 2011a; Maddison and Maddison 2011b). Sequences were aligned, inspected for length variation, and percent sequence difference among taxa calculated in PAUP ver. 4.0b10 (Swofford 2002). Finally, sequences were annotated and uploaded to GenBank at the NCBI website (www.ncbi.nih.gov ).

### Descriptive taxonomy

**Figure 1.**
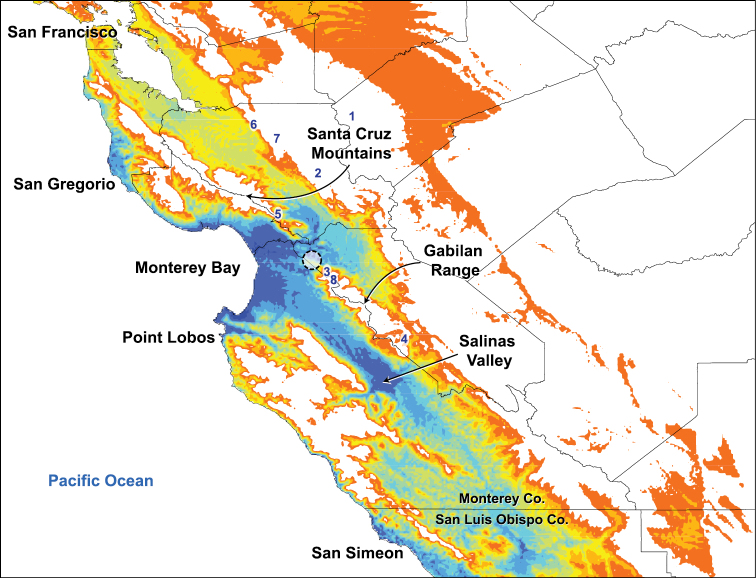
Niche-based distribution model inferred in Maxent. The model indicates predicted habitat suitability for *Illacme plenipes* based on climatic variables extracted from known geographical coordinates of the species. High levels of habitat suitability are denoted in blue and low levels in red (reverse heat map). Coordinates of recently collected specimens are indicated by a circle around the locations (northwest of the Gabilan Range) to preserve the confidentiality of sensitive habitat. Localities surveyed for additional populations of *Illacme plenipes*: **1** Frank Raines Park **2** Henry Coe State Park **3** Fremont Peak State Park **4** Pinnacles National Monument **5** Mount Madonna County Park **6** Alum Rock **7** Joseph D. Grant County Park **8** El Rancho Cienega del Gabilan.

*Illacme plenipes* is represented in natural history museum collections by 17 known specimens, which includes type and non-type material. These specimens were borrowed from the following repositories: Florida State Collection of Arthropods (FSCA), Smithsonian Institution (USNM), and Virginia Museum of Natural History (VMNH). Newly collected material, compared with historical type specimens to confirm species identity, was subsequently georeferenced and databased. The precise locations of recently collected specimens are not plotted on the distribution map; instead, a circle around the coordinates is shown to preserve the confidentiality of sensitive habitat (Fig. 1). Type specimens collected by Cook in 1926 are from an imprecise location on “San Juan grade above Salinas, San Juan Bautista, Calif. Nov. 27, 1926”. However, based on the description, it pro-bably lies on the north side of the Gabilan Range on San Juan Grade Road or Old Stage Road in a radius of 4 km around the coordinates 36.831371°N, -121.562808°W. Due to sensitivity of the habitat and extreme rarity of individuals, locality coordinates from georeferenced material is available upon request from the corresponding author. All of the material (including types and non-type material) was measured, examined in detail and is listed in the “Material examined” section. Specimens were measured at 18 locations on the exoskeleton to summarize continuous morphological variation: (1) body length from anterior margin of labrum to posterior margin of paraprocts, **BL**; (2) head width, **HW**; (3) head length, **HL**; (4) interantennal socket width, **ISW**; (5) antennomere 6 width, **AW**; (6) collum width, **CW**; (7) metazonite width at 1/4 length of body, **W1**; (8) metazonite width at mid-length of body, **W2**; (9) metazonite width at 3/4 length of body, **W3**; (10) metazonite length at 1/4 length of body, **L1**; (11) metazonite length at mid-length of body, **L2**; (12) metazonite length at 3/4 length of body, **L3**; (13) metazonite height at 1/4 length of body, **H1**; (14) metazonite height at mid-length of body, **H2**; (15) metazonite height at 3/4 length of body, **H3**; (16) first apodous metazonite width, **AS1**; (17) anterior gonopod article 5 width, **A5W**; and (18) posterior gonopod article 5 width, **P5W**. Body length was measured from digital photographs of specimens captured through the eyepieces of a Leica M125 stereomicroscope (Wetzlar, Germany) with an iPhone 4 (Apple, Cupertino, CA) using the segmented line measurement tool in ImageJ64 (Rasband 2011). All measurements are recorded in millimeters and these units are omitted throughout the paper. Anatomical measurements in the variation section are given with the following four summary statistics in the following order and format: maximum-minimum (mean/standard deviation). The mean of measurements 7–9 (average body width across three metazonites) is given as “**WM**”; mean of 10–12 is **“LM”** (average metazonite length); and mean of 13–15 is “**HM**” (average body height). The number of segments were counted and number of legs (*l*) then calculated according to the following formula: ***l* = ((*p* + *a*) x 4) – (*a* x 4) – (10)**, where *p* is the number of podous tergites (each with four legs), *a* is the number of apodous tergites (each without legs), and 10 is the number to be subtracted because the first tergite (or the collum) is legless and the second through fourth tergites (the millipede “thorax”) have only two legs apiece. The gonopods, modified leg pairs 9 and 10 are included in the leg count, albeit non-ambulatory. The telson, which is not a segment and does not bear legs (posterior to the proliferation zone), is not included in the formula (Enghoff et al. 1993). Segment architecture for the specimens is denoted by the shorthand ***p* + *a + T***, where *T* is the telson and always 1, however always included in the notation (following Enghoff et al. 1993) to indicate that it is never incorporated in the segment tally. Live material was observed through the eyepieces of a Leica 12.5 stereomicroscope to document *Illacme plenipes* motion, silk production and live habit. Videos were recorded with a Nikon Coolpix 995 digital camera through a C-mounted phototube according to methods described by Marek and Bond (2006). The antennal sensilla nomenclature follows that of Nguyen Duy-Jacquemin (1974) and Chung and Moon (2006). All of the measured material is composed of adult males and females. Because of their rarity, and presumed sensitivity of the species to over-collection, juvenile specimens were not targeted for collection, and are therefore not included in the measurements (one juvenile specimen, listed in the materials examined, was inadvertently collected). Juveniles were identified in the field by a lack of gonopods, small length (≤ 10 mm) and weakly calcified cuticle. Adult males were easily identified by the presence of gonopods, and adult females tentatively by the combination of a lack of gonopods and lengths ≥ 30 mm.

### Data resources

The data underpinning the analysis reported in this paper are deposited in the Dryad Data Repository at http://dx.doi.org/10.5061/dryad.3b3h8 and in the National Center for Biotechnology Information’s genetic sequence database GenBank under the accession numbers: JX962721 – JX962725 (http://www.ncbi.nlm.nih.gov ).

## Results

### Fieldwork

Individuals of *Illacme plenipes* were found at three localities, geographically separated by a maximum of 4.5 aerial km. The first collecting event was on 29 November 2005, the second on 8 December 2005, and the third 16 December 2007. One survey, at which time specimens were found but not collected, occurred 27 January 2006. Each locality is in the northwestern Gabilan foothills no more than 4.5 aerial km from the mission at San Juan Bautista and 3.2 aerial km southwest of the San Andreas Fault. *Illacme plenipes* were not found in any of the other sites investigated. Individuals were found in moist oak-wooded valleys beneath large arkose sandstone boulders (approximate mean mass = 40 kg), clinging to the surface usually about 10 – 15 cm below the top of the soil. *Illacme plenipes* specimens were always found on these boulders and underground, either on the stone surface, in the lacuna between the stone and the soil, or partially imbedded in the soil horizon. Specimens were never found directly on the normally dry bottom of the stones, or on fallen logs or any other decaying organic matter or detritus. *Illacme plenipes* were consistently discovered by closely examining the stone surface (approximately 10–15 cm below soil) and the edge of the crater after removing the stone. Nine additional specimens, comprising 4 males, 4 females, and a juvenile were found throughout 2005–2007 in three localities (increasing the total number of specimens for *Illacme plenipes*, which includes the type series, to 17 total: 6 males, 10 females, and a juvenile). *Illacme plenipes* were uncommon at every locality and individuals were only found after one hour of two persons surveying a suitable-appearing site. Individuals were typically encountered beneath the stones singularly; no more than two individuals were ever found simultaneously.

### Ecological niche modeling

The niche-based distribution model for *Illacme plenipes* indicates the highest probability of occurrence, representing ecological suitability for the species, in the terrestrial areas on the periphery of Monterey Bay extending just past the gap between the Santa Cruz Mountains and Gabilan Range and throughout the Salinas Valley (Fig. 1). Areas of medium to high probability extend from Monterey Bay along a thin region on the coast northward to San Gregorio and southward to Point Lobos. There are other areas of medium to high probability, also restricted to the coast, between San Simeon in the north and the western boundary between Monterey and San Luis Obispo counties.

### DNA barcoding

Polymerase chain reaction of the COI barcoding region, when electrophoresed and visualized on a 12% agarose gel, recovered single bands of uniform lengths in all species. Sanger sequencing resulted in sense/antisense chromatograms reads of ~600 bp in length when contiguous fragments were assembled in Mesquite. Mean Phred quality scores of individual contigs are between 73–80. When aligned and ragged ends trimmed, sequence length is invariant between species. Mean nucleotide percent sequence difference between species is 25% and between amino acid sequences (total difference), 17%. The NCBI GenBank accession numbers are as follows: *Illacme plenipes* (JX962724), *Gosodesmus claremontus* (JX962723), *Brachycybe producta* (JX962721), *Brachycybe rosea* (JX962722), and *Siphonacme lyttoni* (JX962725). The COI barcodes of the Siphonophorida species (*Illacme plenipes* and *Siphonacme lyttoni*) and the Platydesmida species (*Gosodesmus claremontus*, *Brachycybe producta*, and *Brachycybe rosea*) are hitherto the only that exist for these two orders; there is only one other DNA barcode for the entire subterclass Colobognatha. The following species are listed in order of increasing percent nucleotide difference from *Illacme plenipes*, indicated in parentheses (mean percent difference of amino acids proceeds after the “/”): *Gosodesmus claremontus* (28.7% / 23.8%), *Brachycybe producta* (29.7% / 24.4%), *Siphonacme lyttoni* (29.9% / 22.3%), and *Brachycybe rosea* (30.6% / 24.4%).

### Taxonomy. Class Diplopoda de Blainville in Gervais, 1844. Subclass Chilognatha Latreille, 1802/1803. Infraclass Helminthomorpha Pocock, 1887. Subterclass Colobognatha Brandt, 1834. Order Siphonophorida Hoffman, 1980. Family Siphonorhinidae Cook, 1895

#### Genus Illacme

Cook & Loomis, 1928

http://species-id.net/wiki/Illacme

Illacme Cook and Loomis 1928: 12; Chamberlin and Hoffman 1958: 189; Buckett 1964: 29; Jeekel 1971: 39; Hoffman 1980: 116; Shelley 1996b: 23; Shelley 1996a: 1808; Hoffman 1999: 195; Jeekel 2001: 46; Marek and Bond 2006: 707; Shelley 2010: 45.

##### Type species.

*Illacme plenipes* Cook and Loomis 1928: 12; by original designation.

##### Family placement.

**Figure 2–5.**
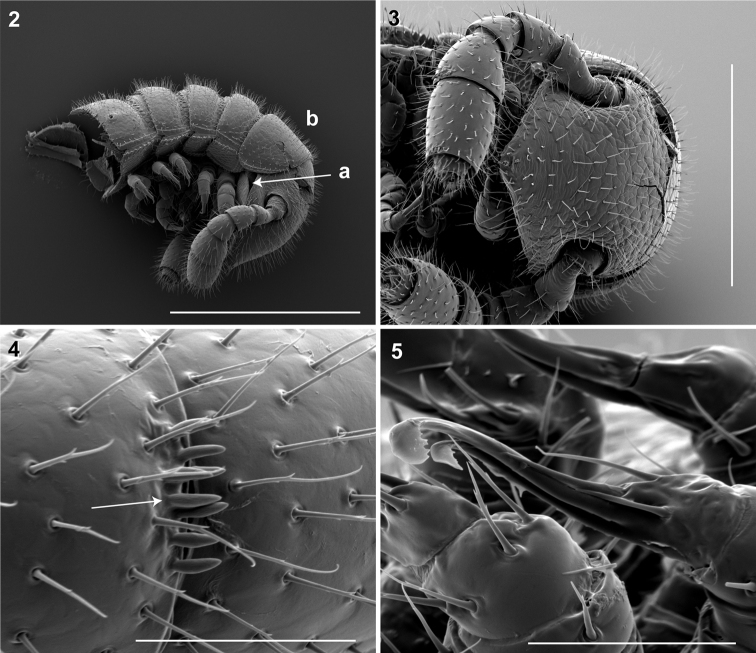
**2** Lateral (right) view of head and segments 1–5 (♂). **a** Lateral opening apparent between gnathochilarium and head capsule; gnathochilarium, mandible and head capsule noticeably separate at base, 1/3 head length distally from mandibular joint **b** Collum not covering head, with straight cephalic edge, gradually tapering laterally. Scale bar 0.5 mm. **3** Ventral view of head, antennae and segments 1 – 5 (♂).Scale bar 0.3 mm. **4** Lateral (right) view of antennomeres 5, 6 (♂).Arrow, small basiconic sensilla (Bs_2_) in cluster of 7 or 8 oriented apical dorsally (retrolaterally) in slight depression on antennomeres 5, 6. Scale bar 0.05 mm. **5** Oblique (right) view of right posterior gonopod (♂).Posterior gonopodal podomere 6 divided, comprising a bundle of 3 stylus-shaped articles. Scale bar 0.05 mm.

**Figure 6.**
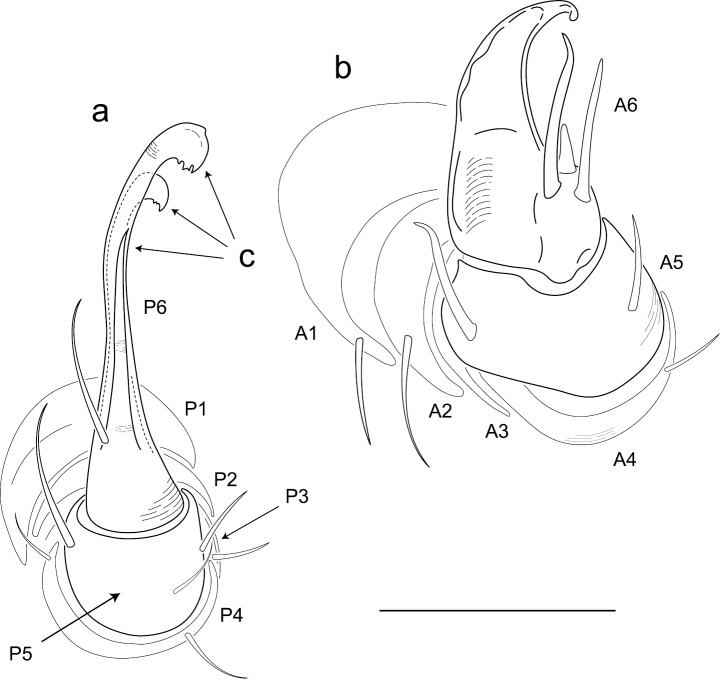
Illustration of anterior and posterior gonopods (♂). **a** Posterior gonopod with podomeres labeled P1-6 **b** Anterior gonopod with podomeres labeled A1-6 **c** 3 stylus-shaped articles. Scale bar 0.05 mm.

*Illacme* is placed with other taxa in the family Siphonorhinidae based on the following characters: Head pear-shaped (♂) or triangular (♀), not elongate or bird beak-shaped, as in the Siphonophoridae (Fig. 2, Morphbank 805574, Appendix I). Antennae elbowed between antennomeres 3, 4 (Fig. 3, Mb-805578). Antennomeres 5, 6 with apical dorsal cluster of 7 or 8 basiconic sensilla (Bs_2_) in slight depression, not deep-set into circular pits, as in the Siphonophoridae (Fig. 4, Mb-805575). Posterior gonopods with distal podomere divided into 2 or 3 branches (Fig. 5, Mb-805576, Fig. 6c). See also diagnoses of *Illacme* in Shelley (1996b, p. 23) and of Siphonorhinidae in Shelley and Hoffman (2004, p. 218).

##### Diagnosis.

**Figure 7.**
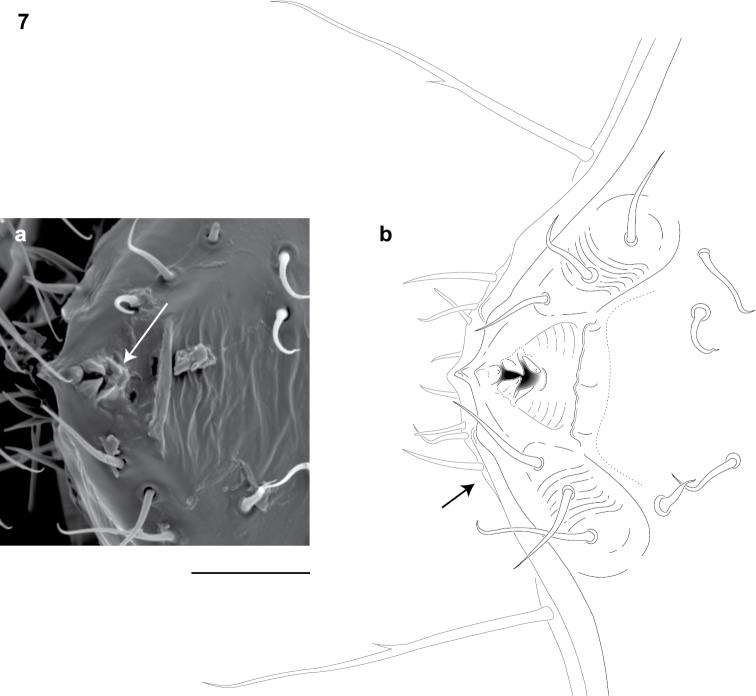
Dorsal view of anterior region of head and labrum (♂). **a** Scanning electron micrograph: arrow, labrum with triangular tooth-lined orifice **b** Line drawing: shaded area, triangular tooth-line orifice; arrow, gnathochilarium. Scale bar 0.02 mm.

**Figure 8.**
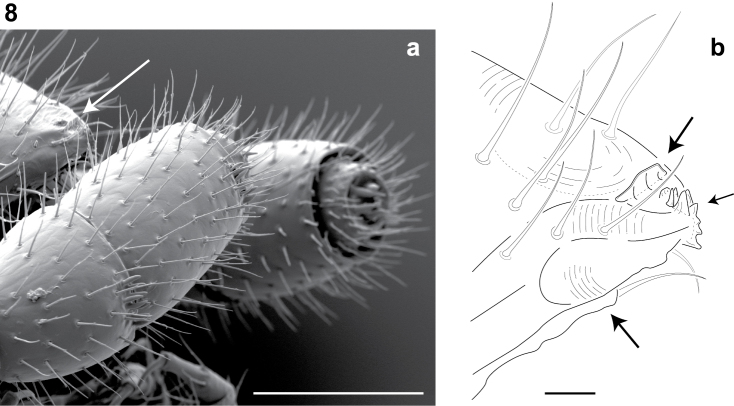
Lateral (right) view of antennal and cephalic apices (♂). **a** Scanning electron micrograph: arrow, denticulate shelf-like carina, projecting dorsally from labrum-epistome margin. Scale bar 0.1 mm **b** Line drawing: top arrow, shelf-like carina; middle arrow, triangular tooth-lined orifice; bottom arrow, gnathochilarium. Scale bar 0.01 mm.

**Figure 9.**
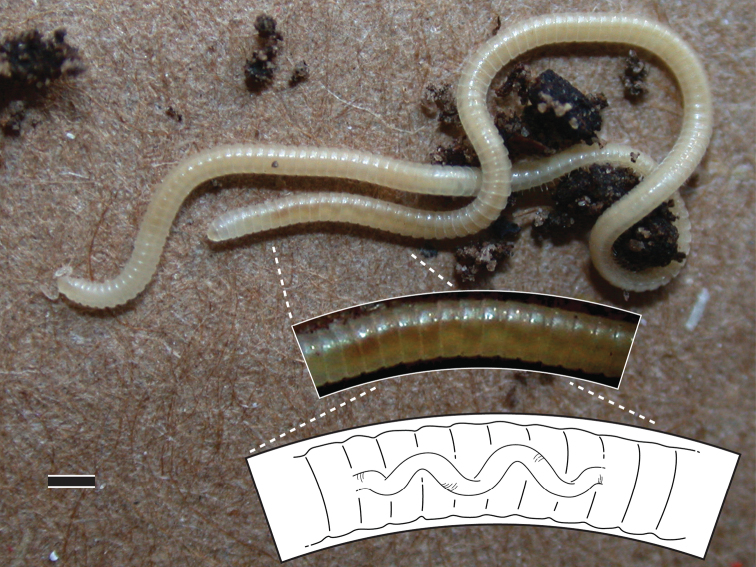
*Illacme plenipes* ♀ with 170 segments and 662 legs (specimen # SPC000931). Top inset, 2× magnified view of posterior segments with corkscrew-shaped metenteron visible through cuticle; bottom inset, 3× magnified illustration of corkscrew-shaped metenteron. Scale bar 1 mm.

**Figures 10–15.**
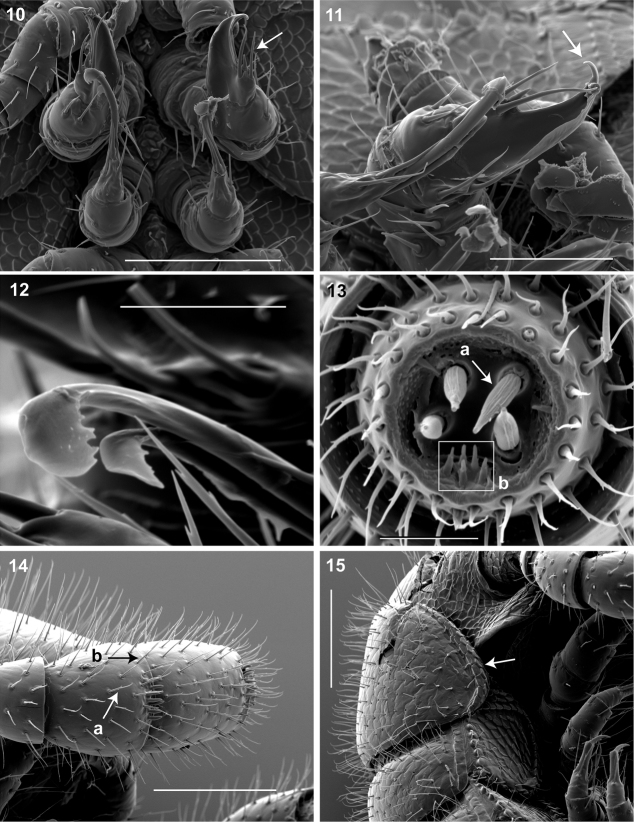
**10** Ventral *in situ* view of gonopods **(**♂**).** Arrow, anterior gonopod thick, more robust than posterior gonopod. Scale bar 0.1 mm **11** Medial view of right gonopods **(**♂**).** Arrow, Anterior gonopodal apex (podomere 6) shovel-shaped; in repose cupped sheath-like around flagelliform posterior gonopodal apex (podomere 6). Scale bar 0.05 mm. **12** Oblique (right) view of right posterior gonopodal apex **(**♂**).** 2 dorsal-most, longest articles laminate distally and recurved laterally, with denticulate posterior margins appearing claw-like. Scale bar 0.02 mm. **13** Antennomere 7 apex **(**♂**)**. **a** Four apical cones (AS) oriented in a trapezoidal cluster on 7th antennomere, with longitudinally grooved outer surface and apical circular invagination **b** Spiniform basiconic sensilla (Bs_3_) in cluster of 5, oriented apical dorsally on 7th antennomere; tips facing apical cones (on longitudinal axis with Bs_2_ on antennomeres 5, 6); each sensillum with 2 barbules. Scale bar 0.02 mm.**14** Lateral (right) view of right antenna **(**♂**)**. **a** Chaetiform sensilla (CS) widely spaced on antennomeres 1-7, each sensillum with 2 or 3 barbules **b** Trichoid sensilla (TS) oriented apically encircling antennomeres 1–7, lacking barbules. Scale bar 0.1 mm. **15** Lateral (right) view of head, collum and segments 2, 3 **(**♂**)**.Arrow, collum with carina present on anterolateral margin, appearing scaly. Carina repeated serially on lateral tergal and pleural margins (absent from telson). Scale bar 0.1 mm.

Adults of *Illacme* are distinct from other siphonorhinid genera (and commonly-encountered millipedes co-occurring with *Illacme plenipes*) based on the combination of – *Exoskeleton*: Body light cream-colored, thread-like, extremely narrow and long (max. width: ♂ 0.55, ♀ 0.64; max. length: ♂ 28.16, ♀ 40.40). Adult individuals with 84 – 192 segments, and with 318 – 750 legs (VMNH paratype ♀ with 192 segments and 750 legs, more than any other organism known on Earth). Body with hirsute vestiture, appearing velvety (Fig. 2, Mb-805577). Antennae elbowed between antennomeres 3, 4 (Figs 2, 3, Mb-805578). Antennomeres 5, 6 enlarged, appearing oversized relative to other millipedes (Figs 2, 3, Mb-805579). Head pear-shaped (♂) or triangular/chevron-shaped (♀), eyeless (Figs 2, 3, Mb-805574, Appendix I). Mouthparts (gnathochilarium, mandibles) and labrum tightly appressed, tapered anteriorly to rounded apex, not bird beak-shaped, as in the Siphonophoridae (Fig. 3, Mb-805586). Labrum with triangular tooth-lined orifice (Fig. 7a, b; Mb-805580). Denticulate shelf-like carina, projecting dorsally from labrum-epistome margin (Fig. 8a, b; Mb-805588). *Internal anatomy*. Posterior one-quarter length of enteron loosely spiraled; when alive, visible through translucent cuticle (Fig. 9, Mb-805582). *Male gonopods*. 9^th^ and 10^th^ leg pairs modified into gonopods, each comprising 6 podomeres (Fig. 6a, b). Anterior gonopod thick, more robust than posterior gonopod (Fig. 10, Mb-805583, Fig. 6b). Anterior gonopodal apex (podomere 6, Fig. 6a, *A6*) shovel-shaped; in repose, cupped sheath-like around flagelliform posterior gonopodal apex (podomere 6, Fig. 11, Mb-805584, Fig. 6b, *P6*). Posterior gonopodal podomere 6 divided, comprising a bundle of 3 stylus-shaped articles (Fig. 5, Mb-805627, Fig. 6a, *P6*); remaining siphonorhinid taxa have 2 stylus-shaped articles with a small spine (*Nematozonium filum*) or 2 articles without a spine (*Siphonorhinus* Pocock, 1894 species and *Kleruchus olivaceus* Attems, 1938). 2 dorsal-most, longest articles of P6 laminate distally and recurved laterally, with denticulate posterior margins appearing claw-like (Fig. 12, Mb-805585, Fig. 6a, *P6*). Ventral-most, shortest article of P6 acuminate distally, spike-like. *Habit in life*. Movement very slow, nearly imperceptible (Appendix II, III). Antennae movement rapid, independent. Terminal antennomeres held flat and rapidly tap substrate and surroundings (Appendix IV).

#### Illacme plenipes

Cook & Loomis, 1928

http://species-id.net/wiki/Illacme_plenipes

Illacme plenipes Cook and Loomis 1928: 12. Chamberlin and Hoffman 1958: 189; Buckett 1964: 29; Shelley 1996b: 23; Shelley 1996a: 1808; Hoffman 1999: 195; Jeekel 2001: 46; Shelley and Hoffman 2004: 221; Marek and Bond 2006: 707; Read and Enghoff 2009: 554; Shelley 2010: 45; Shelley and Golovatch 2011: 26.

##### Material examined.

*Type specimens*: ♂ holotype (USNM), 1♂, 3♀ paratypes (FSCA) and 3♀ paratypes (VMNH)—from United States, California, San Benito County, from “near divide between Salinas and San Juan Bautista” [an imprecise location probably on the north side of the Gabilan Range on San Juan Grade Road or Old Stage Road in a radius of 4 km around the coordinates 36.831371°N, -121.562808°W], 27.xi.1926 (Coll. O.F. Cook). *Non-type specimens*: California, San Benito County: 1♂ (SPC000924), 2♀ (SPC000930, -931), Gabilan Range, San Juan Bautista, 29.xi.2005 (Colls: P. and R. Marek); 3♂ (SPC000932, -933, -934), 1 juvenile (SPC000935), *loc. ibid*., 8.xii.2005, (Coll: J. Bond). 2♀ (SPC001187, MIL0020), Gabilan Range, San Juan Bautista, 16.xii.2007, 13:00 (Colls: P. and R. Marek).

##### Diagnosis.

(See generic diagnosis.)

##### Description of holotype

**Figure 16–21.**
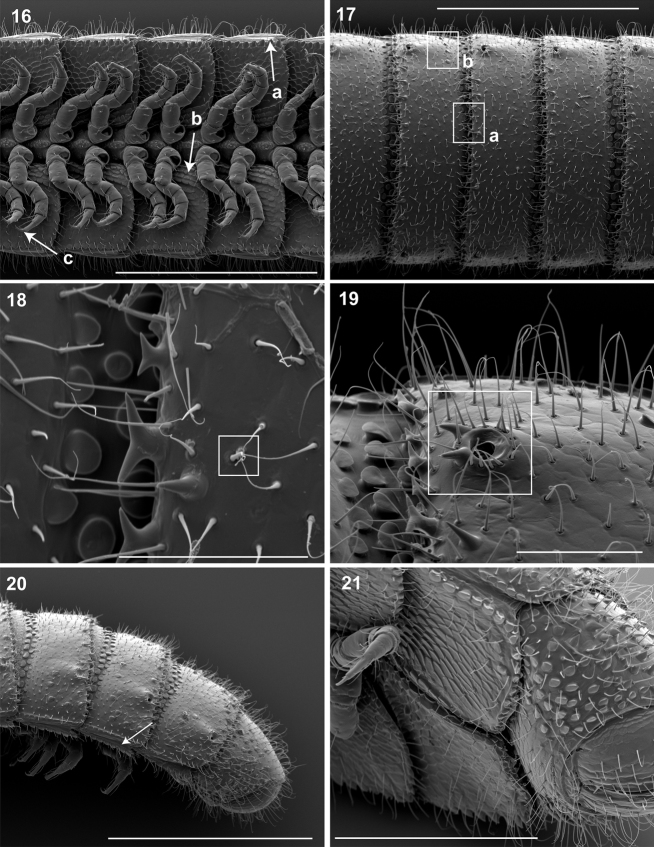
**16** Ventral view of segments (♂). **a** Lateral tergal and pleural carinae jagged, pronounced on midbody segments **b** Pleurite medial margin broad, with scaly carina **c** Postgonopodal tarsus with thinner claw and without bifurcation, but with stout seta. Scale bar 0.4 mm. **17** Dorsal view of segments (♂). **a** Metazonite posterior margin (limbus) lined with posteriorly projecting anchor-shaped spikes and a row of conical spikes just dorsal to anchor-shaped spikes **b** 3 or 4 stout flat tubercles opposite ozopore near anterior margin, lunate arrangement encircling ozopore. Scale bar 0.4 mm. **18** Dorsal view of tergites (♂). Square, tergal setae tipped with silk-like exudate, tangled, appearing adhered to neighboring setae. Scale bar 0.05 mm. **19** Dorsal view of left ozopore (♂).Square, ozopores elevated slightly, with 2 stout posteriorly projecting spines and encircled by 13 – 15 robust setae. Scale bar 0.05 mm. **20** Right lateral view of posterior segments and telson (♂). Arrow, tergites, pleurites and sternites separated by arthrodial membrane. Scale bar 0.4 mm. **21** Oblique (right) ventrolateral view of 2 apodous segments, telson, hypoproct and paraprocts (♂).Apodous segments lacking sterna, pleurites contiguous in midline. Apodous tergites densely setose, covered with unevenly distributed spikes. Scale bar 0.2 mm.

**Figure 22–27.**
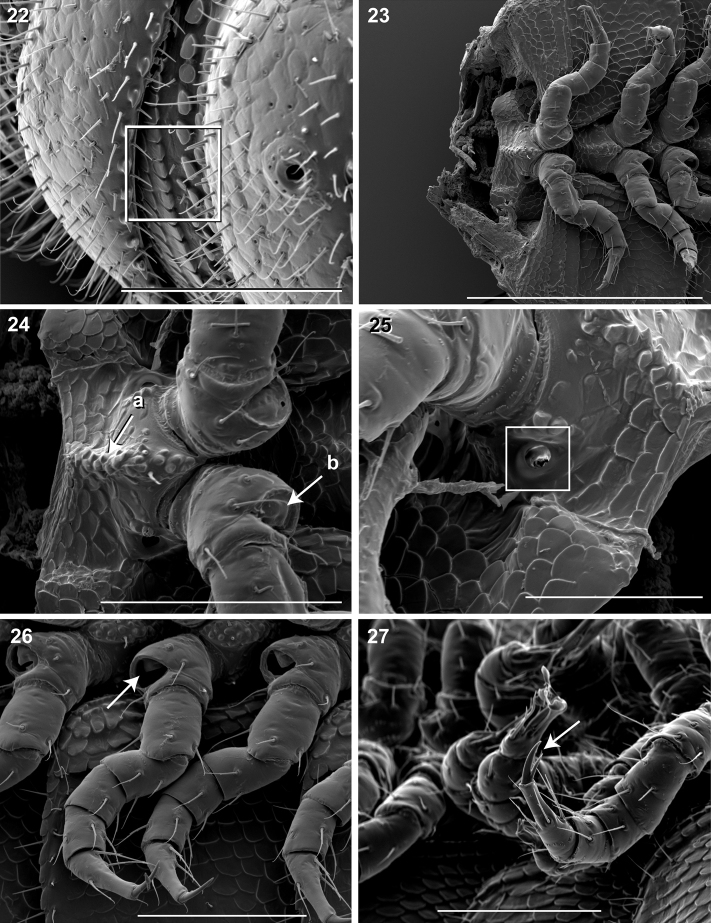
**22** Lateral view of fifth metatergite and prozonite (♂).Square, prozonite highly sculptured, with 5 rows of discoidal flat tubercles; anterior 3 rows staggered and posterior 2 rows aligned. Scale bar 0.1 mm. **23** Ventral view of mid-length sternites, pleurites and legs (♂).Left and right pleurites broadly overlapping sternite, covering spiracles. Scale bar 0.3 mm. **24** Ventral view of mid-length sternites and leg bases (♂). **a** Medial sternal ridge projecting ventrally, with spiracles and legs oriented ventrally **b** Eversible sacs membranous, bulging slightly from opening. Scale bar 0.1 mm. **25** Oblique (right) lateral view of sterna and spiracle (♂). Square, spiracles circular, orifice open; oriented dorsally above legs. Scale bar 0.05 mm. **26**Ventral (right) view of legs, with posteroventral eversible sac opening (♂). Arrow, large posteroventral D-shaped opening for eversible sac. Scale bar 0.1 mm. **27** Oblique (right) lateral view of pregonopodal legs (♂).Arrow, pregonopodal tarsus with stout bifurcate claw. Scale bar 0.1 mm.

(♂) USNM TYPE NO. 976 – *Counts and measurements*: ***p*** = 143. ***a*** = 2. ***l*** = 562. (**143 + 2 + T**). **HW** = 0.30. **HL** = 0.34. **ISW** = 0.20. **AW** = [antennae missing]. **CW** = 0.42. **W1** = 0.53. **W2** = 0.55. **W3** = 0.55. **L1** = 0.20. **L2** = 0.20. **L3** = 0.18. **H1** = 0.31. **H2** = 0.30. **H3** = 0.33. **AS1** = 0.45. **A5W** = 0.05. **P5W** = 0.04. **BL** = 28.16. *Head* pear-shaped, tapered anteriorly to round point at a 160° angle anterior from antennal sockets; occipital area posterior from antennal sockets gradually curved medially towards neck (Figs 2, 3, Mb-805574—note: all SEMs herein are images of specimen #SPC000932, not the holotype). Head pilose, covered with long, slender setae (Fig. 2, Mb-805577). *Mouthparts* (gnathochilarium, mandibles) and labrum tightly appressed, tapered anteriorly to round point (Fig. 3, Mb-805586). Gnathochilarium elements (stipes, promentum, etc.) indistinguishably fused, tightly appressed to the ventral surface of the head, leaving a small opening anteriorly. Lateral opening apparent between gnathochilarium and head capsule (Fig. 2a, Mb-805587). Mandibles thin, stylet-like, with heavily calcified apices (viewed dorsally through translucent head capsule at 400× with a compound microscope). Labrum with triangular tooth-lined orifice (Fig. 7a, b; Mb-805580). Denticulate shelf-like carina, projecting dorsally from labrum-epistome margin (Fig. 8a, b; Mb-805588). Gnathochilarium, mandible and head capsule noticeably separate at base, 1/3 head length distally from mandibular joint (Fig. 2a, Mb-805589). *Antennae* sub-geniculate, elbowed between antennomeres 3, 4, comprising 7 antennomeres (Fig. 3, Mb-805578). Antennae massive distally; antennomeres 5, 6 enlarged (Fig. 3, Mb-805579). Five sensillum types: 4 apical cones (AS) oriented in a trapezoidal cluster on 7th antennomere, with longitudinally grooved outer surface and apical circular invagination (Fig. 13, Mb-805590). Chaetiform sensilla (CS) widely spaced on antennomeres 1-7, each sensillum with 2 or 3 barbules (Fig. 14a, Mb-805591). Trichoid sensilla (TS) oriented apically encircling antennomeres 1–7, lacking barbules (Fig. 14b, Mb-805592). Small basiconic sensilla (Bs_2_) in clusters of 7 or 8; in slight depressions oriented apical dorsally (retrolaterally) on antennomeres 5 and 6; smooth, finger-shaped, 1/2 length of chaetiform sensillum (Fig. 4, Mb-805593). Spiniform basiconic sensilla (Bs_3_) in cluster of 5, oriented apical dorsally on 7th antennomere; tips facing apical cones (on longitudinal axis with Bs_2_ on antennomeres 5, 6); each sensillum with 2 barbules (Fig. 13b, Mb-805594). Antennae extend posteriorly to middle of 3rd tergite. Relative antennomere lengths 6>2>5>3>4>1>7. *Segments*:Collum not covering head, with straight cephalic edge, gradually tapering laterally (Fig. 2b, Mb-805595). Collum with carina present on anterolateral margin, appearing scaly (Fig. 15, Mb-805596). Carina repeated serially on lateral tergal and pleural margins (absent from telson). Lateral tergal and pleural carinae jagged, pronounced on midbody segments (Fig. 16a, Mb-805597). Lateral margin of collum round. *Tergites*: Metazonites rectangular, 3× wider than long, slightly convex (Fig. 17, Mb-805598). Paranota absent. Metazonite dorsal surface pilose, covered with long, slender setae (Fig. 2, Mb-805599). Tergal setae hollow, cavity diameter 1/8 that of setae diameter; tipped with silk-like exudate, tangled, appearing adhered to neighboring setae (Fig. 18, Mb-805600). (NB: Tergal silk-like exudate observed in scanning electron micrographs, and by the observation of fine strands issuing from the metaterga of live individuals, viewed while magnified at 80× with a stereomicroscope. Silk stickiness was indicated by increased adherence of soil particles after handling and live observation of the millipede’s coiled body becoming stuck together.) Metazonite posterior margin (limbus) lined with posteriorly projecting anchor-shaped spikes and a row of conical spikes just dorsal to anchor-shaped spikes (Fig. 17a, Mb-805601). Anchor-shaped spikes alternating in size (large, small) along row. Ozopores oriented dorsally, located near limbus, absent from tergites 1 – 3 and telson. Ozopores elevated slightly (porosteles absent), with 2 stout posteriorly projecting spines and encircled by 13 – 15 robust setae (Fig. 19, Mb-805602). 3 or 4 stout flat tubercles opposite ozopore near anterior margin, lunate arrangement encircling ozopore (Fig. 17b, Mb-805603). Posterior tergites more convex, covered with a greater density of long, slender “silk”-exuding setae (Fig. 20, Mb-805604). Lunate-arranged tubercles opposite ozopores on posterior metazonites: conical and spiked, not flat. Apodous segments lacking sterna, pleurites contiguous in midline. Apodous tergites densely setose, covered with unevenly distributed spikes (Fig. 21, Mb-805605). Telson densely covered with irregularly oriented and unevenly distributed stout spines; posterior margin lined with variably-shaped posterodorsally oriented anchor-shaped spikes. Tergal tubercles and spikes: consistently projecting posteriorly, occasionally posterodorsally. Prozonite highly sculptured, with 5 rows of discoidal flat tubercles; anterior 3 rows staggered and posterior 2 rows aligned (Fig. 22, Mb-805606). *Pleurites* quadrate, flat, with jagged scaly lateral, posterior and medial margins (Fig. 16, Mb-805609). Pleurite medial margin broad, with scaly carina (Fig. 16b, Mb-805610). Left and right pleurites plate-like, comprising 4/5’s of ventral segment space. Left and right pleurites broadly overlapping sternite, covering spiracles (Fig. 23, Mb-805612). *Sternites* free, separate from pleurites; heart-shaped, wider anteriorly. Sternal surface with broad, jagged scales. Medial sternal ridge projecting ventrally, with spiracles and legs oriented ventrally (Fig. 24, Mb-805614). Spiracles circular, orifice open; oriented dorsally above legs (Fig. 25, Mb-805615). Anterior and posterior sternites separate. Tergites, pleurites and sternites separated by arthrodial membrane (Fig. 20, Mb-805616). Arthrodial membrane between tergites and pleurites wider posteriorly. *Telson* pilose, covered with long, slender posteriorly recurved setae (Fig. 20, Mb-805628). Paraprocts semihemispherical, anterior margins slightly scaly. Epiproct absent. Hypoproct small, one-eighth area of paraproct, with row of posterior projecting setae. *Legs*: six subequally shaped podomeres, with coxa slightly shorter and tarsus slightly longer. Legs with sparse setae, appearing similar to trichoid sensilla, with 2 or 3 barbules. Coxae nearly contiguous medially, separated by thin sternal ridge. Large posteroventral D-shaped opening for eversible sac (Fig. 26, Mb-805618). Eversible sacs membranous, bulging slightly from opening (Fig. 24b, Mb-805620). Pregonopodal tarsus with stout bifurcate claw; dorsal subdivision thicker, more arcuate (Fig. 27, Mb-805621). Postgonopodal tarsus with two separate claws, co-terminal on tarsal apex; dorsal claw thick and arcuate, ventral claw thin and setiform (Fig. 16c, Mb-805623). 2nd leg pair with posteriorly oriented coxal gonapophyses; rounded, protuberant, one-third length of prefemur. *Gonopods*: 9^th^, 10^th^ leg pairs modified into gonopods, each comprising 6 podomeres (Fig 6a, b). Anterior gonopod thick, more robust than posterior gonopod (Fig. 10, Mb-805583, Fig. 6b). Anterior gonopodal apex (podomere 6) shovel-shaped; in repose cupped sheath-like around flagelliform posterior gonopodal apex (podomere 6, Fig. 11, Mb-805584). Posterior gonopodal podomere 6 divided, comprising a bundle of 3 stylus-shaped articles (Fig. 5, Mb-805627, Fig 6a). 2 dorsal-most, longest articles of P6 laminate distally, recurved laterally, denticulate posterior margins, appearance similar to a chicken foot in *rigor mortis* (Fig. 12, Mb-805585, Fig 6a). Ventral-most, shortest article of P6 acuminate distally, spike-like. Thin ridge-shaped sterna present between left and right gonopods, thicker between anterior gonopods.

##### Description of largest paratype

(♀) VMNH – *Counts and measurement*s: ***p*** = 190. ***a*** = 2. ***l*** = 750. (**190 + 2 + T**). **HW** = 0.37. **HL** = 0.44. **ISW** = 0.30. **AW** = antennae missing. **CW** = 0.44. **W1** = 0.58. **W2** = 0.58. **W3** = 0.57. **L1** = 0.23. **L2** = 0.21. **L3** = 0.23. **H1** = 0.46. **H2** = 0.44. **H3** = 0.48. **AS1** = 0.44. **BL** = 40.40. Anatomical description similar to male holotype. In combination with its measurements, the following structures *differ* from male holotype. *Head* triangular, chevron-shaped, tapered anteriorly to round point at a 135° angle anterior from antennal sockets; occipital area posterior from antennal sockets straight, not curved medially towards neck. *Cyphopods* large, area 1/6 the segmental area in widest cross-section; almond-shaped, bivalvular, narrow apex oriented ventrolaterally. Valves transparent, glassy. Ventral valve thickened and clam-like, with 4 or 5 thick setae; dorsolateral valve thin and flat, with 2 or 3 spines. Oviduct connected posteriorly to cyphopod, opening oriented ventromedially and located between valves. Oviduct tube wrinkled, appearing highly expandable in width, cross-section 1/8 area of cyphopod. Receptacle, suture and operculum absent.

##### Etymology.

Cook and Loomis (1928) named this species “in highest fulfillment of feet”. *Il* = “in” (Latin); *acme*, άκμή (Greek) = “the highest point, or culmination”; *pleni* = “full” (Latin); *pes* = “foot” (Latin).

##### Variation.

There is negligible variation in coloration among live specimens. (FSCA paratype specimens that have been stored in alcohol for 86 years are dark mahogany brown, which is likely an unnatural color and a result of alcohol preservative, vial stopper and age.) The predominant source of variation between specimens is segment and leg counts (Tables 1–3). Females have between 486-750 legs with a standard deviation of 78, and males between 318–562 legs with a standard deviation of 107. The segments of *Illacme plenipes* (males and females) are uniform in length, width and height along the trunk, and are slightly taller, and more convex, in posterior segments—potentially to accommodate the spiraled metenteron.

**Table 1.** Segment and leg count, head measurements.

	**p**	**l**	**HW**	**HL**	**ISW**	**AW**	**CW**
♂	**84–145** (107/27)	**318–562** (410/107)	**0.295–0.308** (0.301/0.006)	**0.344–0.406** (0.382/0.024)	**0.172–0.202** (0.189/0.011)	**0.098–0.103** (0.101/0.002)	**0.374–0.422** (0.393/0.019)
♀	**126–192** (159/20)	**486–750** (619/78)	**0.308–0.369** (0.335/0.020)	**0.408–0.556** (0.446/0.045)	**0.185–0.295** (0.217/0.033)	**0.098–0.113** (0.103/0.006)	**0.407–0.472** (0.431/0.021)

**Table 2.** Width and length measurements.

	**W1**	**W2**	**W3**	**WM**	**L1**	**L2**	**L3**	**LM**
♂	**0.437–0.526** (0.485/0.033)	**0.467–0.554** (0.500/0.036)	**0.455–0.545** (0.488/0.034)	0.491/ 0.032	**0.148–0.203** (0.173/0.021)	**0.150–0.197** (0.162/0.020)	**0.140–0.183** (0.159/0.017)	0.165/0.019
♀	**0.520–0.620** (0.564/0.035)	**0.531–0.640** (0.569/0.037)	**0.517–0.610** (0.559–0.032)	0.564/0.034	**0.172–0.228** (0.195/0.018)	**0.176–0.209** (0.194/0.012)	**0.157–0.234** (0.194/0.021)	0.194/0.017

**Table 3.** Height, apodous segment/gonopodal width, body length measurements.

	**H1**	**H2**	**H3**	**HM**	**AS1**	**A5W**	**P5W**	**BL**
♂	**0.273–0.400** (0.350/0.057)	**0.277–0.418** (0.337/0.055)	**0.295–0.381** (0.336/0.036)	0.341/0.047	**0.394–0.445** (0.423/0.022)	**0.047–0.055** (0.051/0.003)	**0.036–0.043** (0.040/0.003)	**13.368–28.156** (19.251/6.305)
♀	**0.220–0.486** (0.365/0.077)	**0.289–0.488** (0.384/0.064)	**0.295–0.504** (0.370/0.079)	0.373/0.071	**0.412–0.482** (0.451/0.024)	-	-	**24.541–40.399** (31.055/5.474)

##### Natural history.

**Figure 28.**
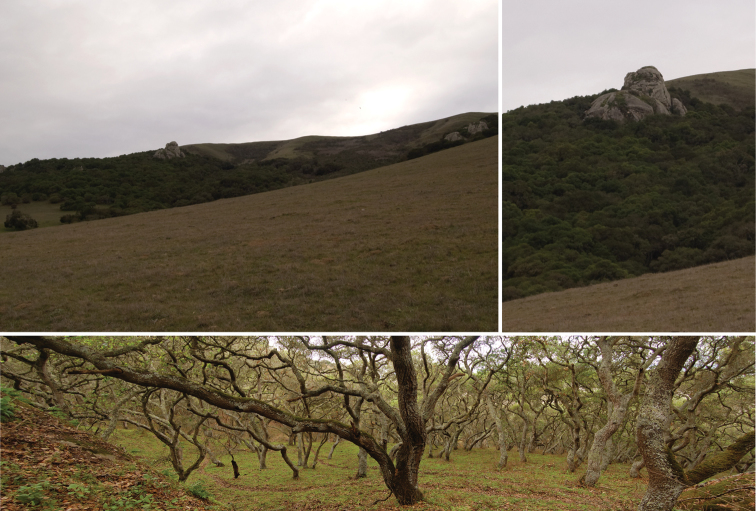
Habitat of *Illacme plenipes*. Top left, view of oak forest where *Illacme plenipes* were encountered. Top right, close up of oak forest and sandstone pinnacle where *Illacme plenipes* occur. Bottom, landscape view of oak forest, cattle trails evident (composite stitched image of three photos, image sides slightly distorted).

**Figure 29.**
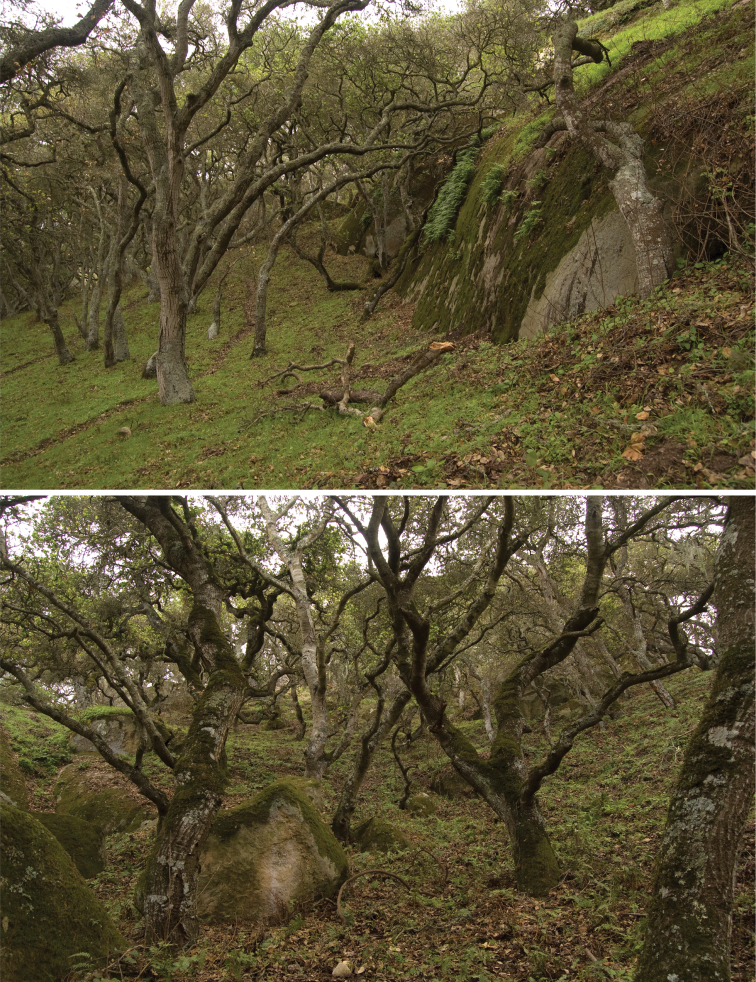
Oak forest understory habitat of *Illacme plenipes*. Top, base of sandstone pinnacle (from Fig. 28), where specimens were found. Bottom, mossy oak forest—close-up of habitat where *Illacme plenipes* individuals were encountered.

**Figure 30.**
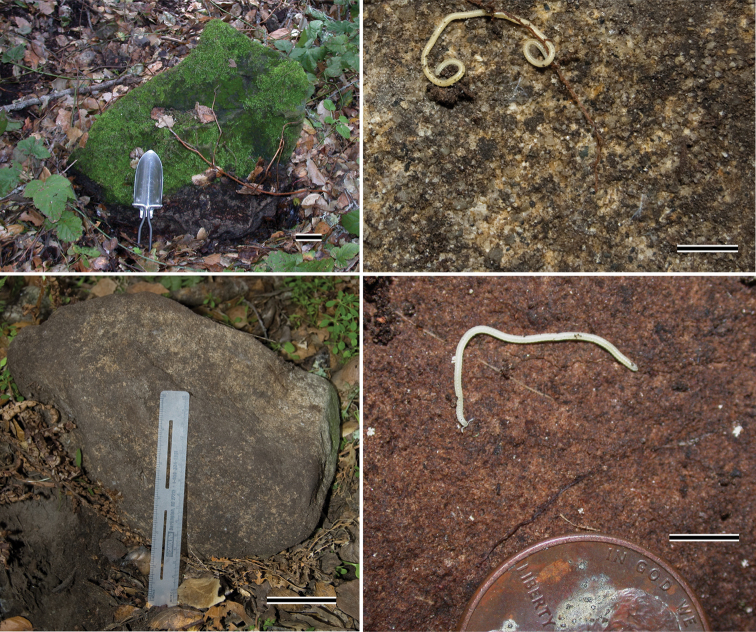
Sandstone microhabitat of *Illacme plenipes*.Top left, 50 kg sandstone from 29.xi.2005 rediscovery locality of *Illacme plenipes*; one ♀ with 666 legs was discovered from beneath the stone (scale bar = 5 cm, hand shovel shown for scale). Bottom left, 30 kg sandstone from the 16.xii.2007 locality, two ♀ (specimen #s: SPC001187, MIL0020) were discovered below the stone (scale bar = 5 cm, 15 cm ruler shown for scale). Top right, surface close up of sandstone from 16.xii.2007 locality with ♂ *Illacme plenipes*, not collected (scale bar = 5 mm). Bottom right, surface close up of sandstone from 29.xi.2005 locality with ♂ *Illacme plenipes* (specimen #: SPC000924, scale bar = 5 mm). Millipedes shown in right two pictures were found clinging to the surface of the stone.

**Figure 31.**
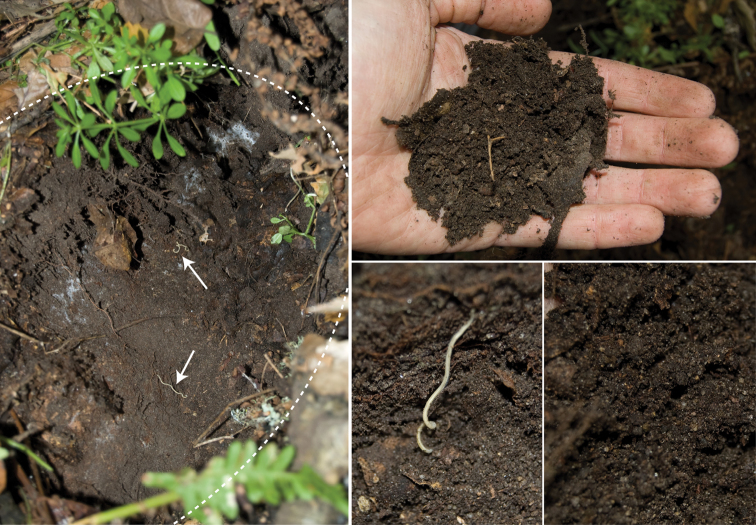
Subterranean soil microhabitat of *Illacme plenipes*. Left, sandstone crater; dotted line indicates crater’s edges, arrows indicate two ♀ *Illacme plenipes* shown *in situ* upon removal of stone (specimen #s: SPC001187, MIL0020—lower individual with anterior trunk segments embedded in soil, upper individual with middle segments embedded in soil). Bottom middle, close up of lower individual from left image. Top right, dark sandy soil from microhabitat. Bottom right, close up of soil showing sandy grain structure.

*Illacme plenipes* specimens were collected during the day in a small valley adjacent to cattle pasture. The woodland habitat was primarily composed of California live-oak, *Quercus agrifolia* (Fig. 28). Understory flora included ferns (bracken, *Pteridium aquilinum*; California polypody, *Polypodium californicum*; and California maiden-hair, *Adiantum jordanii*), California blackberry (*Rubus ursinus*), and poison oak (*Toxicodendron diversilobum*) (Fig. 29). Specimens were found beneath large moss-covered boulders, typically with a mass > 30 kg (Fig. 30). The millipede *Tylobolus uncigerus* (Wood, 1864) (order Spirobolida) was found co-occurring with *Illacme plenipes* at this locality. Other arthropods encountered include: *Aptostichus* and *Calisoga* trapdoor spiders (Mygalomorphae), *Evalljapyx* (Diplura), and *Promecognathus* ground beetles (Carabidae). *Edaphic setting*: Specimens collected in 2007 were found beneath a large stone (Fig 30, about 30 kg). When the stone was removed, individuals were seen corkscrewing outward into the cavity from the soil (Fig. 31). The soil, consisting of moist small-grained substrate, was dark chocolate brown in coloration and somewhat sandy (Fig. 31). The soil did not contain clay particles and seemed to drain water quickly. During the 16 December 2007 collections, soil moisture extended 15 cm below the surface.

##### Distribution.

*Illacme plenipes* is only known from a small area, ca. 4.5 km in diameter, in the northwestern foothills of the Gabilan Range in San Benito County, California.

## Discussion

### “The acme of plentiful feet”

The pattern by which *Illacme plenipes* add segments and subsequently legs post-embryonically between developmental stadia is referred to as anamorphosis (Enghoff et al. 1993). Based on the large number of legs and considerable variation in leg and segment count among adults, anamorphosis likely continues for an indeterminate period, extending well beyond the attainment of sexual maturity (Enghoff et al. 1993; Marek and Bond 2006). Millipedes generally use their numerous legs to burrow between and through obstacles that they encounter (Hopkin and Read 1992; Manton 1954). A leg pair acts to push and propel the myriapod forward, and with two leg-pairs per segment (diplosegments in millipedes represent a fusion of two primordial segments), millipedes create a stronger thrust for a relatively compact body. Millipedes with heavily calcified cuticles and rather incompressible bodies composed of rigid rings (e.g., the Spirobolida and Spirostreptida), burrow through the soil by brute leg force, ramming and bulldozing with a smooth rounded head and collum. In contrast, many millipedes with flexible cuticles and compressible bodies, which are composed of free sternites and pleurites (e.g., other Siphonophorida and *Illacme plenipes*), move through the soil by squeezing flexible anterior segments forward by leg force and subsequently telescoping posterior segments forward and repeating, i.e. the borer millipedes (Hopkin and Read 1992; Manton 1961). The anterior segments in these millipedes are tapered, most noticeably in the Polyzoniida, and bore and wedge to facilitate movement through the soil. With *Illacme plenipes*, the numerous legs presumably impart greater motive force to push within a subterranean microhabitat, and to cling tightly to the surface of sandstone boulders (as described below).

### Natural history

The diet of *Illacme plenipes* is unknown. Given the shape of its mouthparts, the typical millipede diet in which decaying organic matter is mechanically fragmented is unlikely for the species. *Illacme plenipes* possesses a comb-like structure on the posterior margin of the labrum and an open triangular tooth-lined “mouth” formed by an orifice though the labrum (Fig. 7a, b; Fig. 8a, b; Mb-805580; Mb-805588). The mouthparts are composed of the stylet-like mandibles and the gnathochilarium (structures observed between 500-2000× with a scanning electron microscope and the mandibles through the translucent head capsule at 400× with a compound microscope). These mouthparts are tightly appressed and tapered anteriorly to a rounded point. Given that the mandibles appear stylet-like, and assuming the mouthparts are moveable, a functional hypothesis for feeding is that the gnathochilarium hinges open, the mandibles are protruded to pierce plant and/or fungal tissue, and then the tooth-lined mouth is used to suck out the fluid contents. The teeth and labral comb could serve to filter particulates exceeding a certain size. Other Colobognath millipedes with somewhat reduced mouthparts, for example species of the family Andrognathidae, feed on fungus or other live plant or soft organic matter (Gardner 1974). Manton (1961) described the feeding of captive siphonophorids *Siphonophora portoricensis* Brandt, 1837 and *Siphonophora* (=*Siphonocybe*) *hartii* (Pocock, 1894) and observed individuals probing decayed vegetation with their beaked proboscises, after tapping the material with their antennae. Fungi were not observed associated with *Illacme plenipes*, as they are often with species of Platydesmida. However, live plant tissues, especially fine grass roots that are often confused with *Illacme plenipes*, were abundant where specimens were encountered and are a potential food source. The enteric anatomy of *Illacme plenipes* indicates a water or nutrient-poor diet. Individuals of the species possess a regularly spiraled metenteron, which is similar to glomeridan millipedes and a diverse range of animals (e.g., snails and loricariid catfish with spiraled digestive tracts). A spiraled metenteron coupled with the extreme number of segments lengthens the digestive tract and hence the body. This lengthening might function to increase the absorptive surface area in order to extract maximum benefit from a water or nutrient-deficient diet. (It is uncertain whether the spiraling is restricted to the metenteron, a structure concerned with water resorbtion via the Malphigian tubules, or a combination of the metenteron and mesenteron.) Alternatively, a long trunk may function to store additional eggs, and potentially evolved under fecundity selection. Consistent with this hypothesis, *Illacme plenipes* are sexually size dimorphic: female maximum length (BL) and maximum width (BM) is 1.43-fold and 1.16-fold greater than male length and width.

Based on natural history observations of *Illacme plenipes* in the field, individuals are always found approximately 10 – 15 cm beneath the soil, or clinging to the surface of large sandstones. The great number of legs may benefit a deep subterranean lifestyle clinging to sandstone. *Illacme plenipes* has bifurcate claws on anterior legs and two separate claws, coterminal on the tarsal apex (in lieu of the abifurcation), on posterior legs. In several millipede species, e.g. *Cylindroiulus fimbriatus* Enghoff, 1982 and *Dolistenus savii* Fanzago, 1874, the additional claws serve a stone-clinging function for surface adherence and an epilithic lifestyle (Enghoff 1983; Manton 1961). *Illacme plenipes* has large eversible sacs, structures that have also been implicated in surface clinging in petrophilic colobognath millipedes (Manton 1954; 1961). On the dorsal surface of the millipede, setae secrete a silk-like substance, which appears sticky, and may be used for clinging to the stone surface. The secretions seem to increase with handling, perhaps alternatively indicating an anti-predatory function (Shear 2008; Youngsteadt 2008). The silk may also function as a soil shedding mechanism to allow efficient burrowing, or as a means to ensnare parasites or debris particles (Youngsteadt 2008). The chemical composition of the silk is unknown. While millipedes in seven other orders of Diplopoda produce a silk-like substance from various body structures, its threads are not true silk composed of protein (one order produces silk from openings on the legs, one order from metatergal setae like *Illacme plenipes*, 4 orders from epiproctal spinnerets, and one order from both metatergal and epiproctal setae). In contrast with the silk’s origin from the setal tip in *Illacme plenipes* (Fig. 18, Mb-805600), the other seven orders appear to produce silk from pores located at the setal base (Shear 2008). The diverse locations where silk originates in millipedes (legs, epiprocts, metatergal setae), suggests independent origins and precludes homology (Shear, 2008). The extrusive sticky appearance of *Illacme plenipes*’silk-like secretion may indicate a mucopolysaccharide identity, as is the composition of epiproctal silk spun by millipedes in the order Polydesmida (Adis et al. 2000; Shear 2008).

In contrast with the smooth exoskeleton of the bulldozer millipedes, *Illacme plenipes*’ has a multiplicity of projections and cuticular ornaments including anchor-shaped spikes, discoidal tubercles, long silk-secreting setae and jagged body plates. Several of these projections (e.g., the peculiar anchor-shaped spikes — Fig. 17a, Mb-805601) have been documented in other taxa in the Siphonophorida and Julida (Akkari et al. 2011; Read and Enghoff 2009). In a survey of Siphonophorida from Brazilian collections, Read and Enghoff (2009, Fig. 4) provide SEMs that document an individual with similar appearing tergal sculpture, including anchor-shaped spines, discoidal metatergal tubercles, long (possibly silk-secreting) setae, and two shape classes of prozonital tubercles. The prozonital microsculpture of *Illacme plenipes* also appears to correspond in shape and location with several taxa of Polydesmida (Akkari and Enghoff 2011; Mesibov 2012). In the Polydesmida, like *Illacme plenipes* (and other taxa in the Siphonophorida), the prozonital microsculpture is divided into two shape classes: a smooth scaly texture anterior to the prozonital transverse ridge and a rugged knobby surface, with discoidal tubercles or spherical knobs posterior to the ridge. The presence of spherical knobs and other cuticular ornaments in certain families of Polydesmida appear to reflect major evolutionary groups in the order (Akkari and Enghoff 2011). The function of the cuticular ornaments in *Illacme plenipes* is uncertain. Authors have suggested several hypotheses for the function of various projections including a locking mechanism for volvation, in the case of the anchor-shaped spike in Julida, and maintaining a cloak of soil for camouflage, in the case of branching tree-shaped setae in Polydesmida (Shear, 1977).

### Evolutionary relationships

The widely scattered distribution of modern Siphonorhinidae, predominately in the Southern Hemisphere except with *Illacme plenipes* in North America, indicates that their most recent common ancestor likely predates the breakup of Pangaea more than 200 million years ago. A phylogeny for Siphonorhinidae, or any taxa in the four orders of Colobognatha, does not exist, except for a recent species phylogeny of the genus *Brachycybe* in the order Platydesmida (Brewer et al. 2012). Even though the number of COI barcodes for the Colobognatha is low and the region may not be ideal for recovering the ancient divergences between the colobognath taxa represented here (likely > 200 mya), we inferred a preliminary phylogeny with the COI nucleotides using a maximum likelihood tree search in RAxML ver. 7.0.3 (Stamatakis 2006). We recovered monophyletic Platydesmida and Siphonophorida with *Siphonacme lyttoni* sister to *Illacme plenipes*. When *Polyzonium germanicum* (Polyzoniida) was included in the RAxML analysis and visualized in an unrooted tree, it occurred on an intervening branch between Siphonophorida and Platydesmida clades. (*Polyzonium* COI barcoding sequences from Spelda et al. 2011).

The paleoendemic species *Illacme plenipes* is the sole representative of the family in the Western Hemisphere. Remaining genera in the family occur primarily in the Old World tropics in Wallacea, Sundaland, Himalayas (*Siphonorhinus* species), Indo-Burma (*Kleruchus olivaceus* and *Siphonorhinus* species), and Maputaland-Pondoland-Albany (*Nematozonium filum*). The closest relative of *Illacme plenipes* is uncertain. The present day range of Siphonorhinidae may be the remnant of an ancient and widespread tropical distribution across Pangaea. The most likely sister taxon to *Illacme plenipes* is *Nematozonium filum* from South Africa, as they share a number of anatomical similarities. Among the known species of Siphonorhinidae, a South African species is a probable candidate for closest relative based on other close relationships between co-distributed taxa, for example the flightless Californian beetle genus *Promecognathus* and its close relatives in the tribe Axinidiini in South Africa (Erwin 1985; McKay 1991). *Nematozonium filum* and *Illacme plenipes* share posterior gonopods divided into 2-3 thin articles (three in *Illacme plenipes* and two plus a small spine in *Nematozonium filum*), and each millipede is very long and spindly (Attems 1951; Shelley and Hoffman 2004). Known *Illacme plenipes* specimens compose a maximum of 192 segments and *Nematozonium filum*, 182 segments. (However, some species of the family Siphonophoridae also reach beyond 182 segments, e.g., *Siphonophora millepeda* Loomis, 1934 with 190 segments). Individuals of *Kleruchus olivaceus* and *Siphonorhinus* species have bifurcate posterior gonopods (i.e. without a spine as in *Nematozonium filum*), fewer segments, and a shorter and more compact body form. Siphonorhinid millipedes, studied sporadically over the last 80 years by different taxonomists concentrating on various geographic faunas, are ideal candidates for a modern synthesis and molecular phylogenetics. For example *Siphonorhinus*, as is certainly the case for *Siphonophora*, seems to be a taxonomic dumping ground for long and spindly Siphonophorida without a bird-like beak or paranota (Jeekel 2001). The diversity of anatomical forms in the Siphonophorida, in particular the Siphonorhinidae, is quite conserved compared to other diplopod taxa. Compared to other Colobognatha, somatic anatomical diversity across lineages is low and indicates that early Siphonophorida may have appeared similar to present day species. This suggests that contemporary habitats, and current environmental factors affecting body shape, may have been similar to those in which early Siphonophorida taxa occurred. *Illacme plenipes* and related lineages may have persisted unchanged in a mild, constant habitat for hundreds of millions of years. This idea raises fascinating questions about climate and habitat constancy where Siphonorhinidae occur (its six regions also happen to be global biodiversity hotspots), and also important concerns about the conservation of the species and co-inhabitants that may have persisted in these mild climates that are now currently threatened by global climate change.

### Local biogeography

The influence of the marine layer and thick inland fog, which creates a unique climate for the area, may have contributed to a stable environment for *Illacme plenipes*. Areas with high probability of occurrence (Fig. 1) also receive a frequent layer of fog (Johnstone and Dawson 2010). The fog extends into the Monterey Basin and Salinas Valley and is nearly superimposable with the area of highest probability of occurrence on the DM (Appendix V). Rainfall is very seasonal where *Illacme plenipes* occurs, falling predominately between the months of November and March (when individuals were encountered). Cool, wet winters are punctuated by warm, dry summers when the habitat is much drier, and soil beneath stones is nearly devoid of moisture. Although surveys were not conducted during the summer, individuals are less likely encountered at this time, and probably in a reduced state of activity deep underground. Of the nine localities specifically surveyed for additional populations of *Illacme plenipes*, only one, the ranch locality near San Juan Bautista, housed a second population. These localities were initially chosen according to similarity with the 2005 locality near San Juan Bautista, and not as a result of the DM that was constructed for this study. All of the localities searched were indicated as low probability in the DM except Alum Rock, where specimens were not found, and the San Bautista ranch. Habitat suitability may be influenced by the presence of fog and/or the particular edaphic conditions and geology of the localities. Niche-based distribution modeling typically does not include edaphic factors or the geology of the area, and individuals of *Illacme plenipes* were always found in areas with arkose sandstone. The three habitats where individuals were encountered overlay marine arkosic sandstone deposits between the Vergeles and San Andreas faults (Dibblee et al. 1979). High probability of *Illacme plenipes* occurrence is also present in the areas around the southern Monterey Bay and Salinas Valley that overlay more recent surficial alluvial deposits. While the probability of occurrence is high in these unsampled areas, the edaphic setting indicates lower suitability. The soils of the Monterey Basin and Salinas Valley are composed of alluvial sediments and fine-grained deposits, lacking the large arkose sandstones and boulders that *Illacme plenipes* may be specially adapted to. Nonetheless, there is a present-day low overall probability of occurrence of *Illacme plenipes* in the area, or of any other native soil dweller for that matter, since the Salinas Valley is heavily influenced by agriculture and development.

### Conservation

*Illacme plenipes* is threatened by extinction as a result of its restricted geographical distribution, narrow microhabitat requirements, seasonal rarity, and low observed population numbers. Natural populations are threatened by habitat loss due to rampant development and intense land use in the area (agricultural, industrial, transit and housing), climate change, invasive species, and potential for over-collecting. The restricted location of *Illacme plenipes*, limited to the gap between the Santa Cruz Mountains and Gabilan Range at the eastern fog limit, may be due to edaphic requirements (soils composed of sandstone or other native formations in the area: San Lorenzo Formation or Dacitic volcanic rocks), or extirpation due to the heavy agricultural influence around Monterey Basin and the Salinas Valley since the 1800s. In contrast with habitat degradation from development and farming, the presence of cattle does not appear to negatively affect *Illacme plenipes*. At each locality where *Illacme plenipes* was discovered, there was noticeable influence of cattle on the habitat. Boulders under which *Illacme plenipes* occurred were sometimes a meter away from deep cattle hoof prints. The most serious impacts that *Illacme plenipes* faces are human-induced habitat loss and climate change. As suggested by the distribution model and *Illacme plenipes*’ apparent dependence on marine layer fog (likely influencing moisture and stability of its habitat), the documented 33% reduction in coastal California fog due to higher atmospheric and ocean temperature since the early 1900s (Johnstone and Dawson 2010) may severely impact the species and hasten its extinction. The few locations where *Illacme plenipes* exist are unique storehouses of this evolutionary relict, and potentially other ancient lineages that await discovery.

## Morphbank annotations

(Published at www.morphbank.net ):

http://www.morphbank.net/?id=805574

http://www.morphbank.net/?id=805575

http://www.morphbank.net/?id=805576

http://www.morphbank.net/?id=805577

http://www.morphbank.net/?id=805578

http://www.morphbank.net/?id=805579

http://www.morphbank.net/?id=805580

http://www.morphbank.net/?id=805582

http://www.morphbank.net/?id=805583

http://www.morphbank.net/?id=805584

http://www.morphbank.net/?id=805585

http://www.morphbank.net/?id=805586

http://www.morphbank.net/?id=805587

http://www.morphbank.net/?id=805588

http://www.morphbank.net/?id=805589

http://www.morphbank.net/?id=805590

http://www.morphbank.net/?id=805591

http://www.morphbank.net/?id=805592

http://www.morphbank.net/?id=805593

http://www.morphbank.net/?id=805594

http://www.morphbank.net/?id=805595

http://www.morphbank.net/?id=805596

http://www.morphbank.net/?id=805597

http://www.morphbank.net/?id=805598

http://www.morphbank.net/?id=805599

http://www.morphbank.net/?id=805600

http://www.morphbank.net/?id=805601

http://www.morphbank.net/?id=805602

http://www.morphbank.net/?id=805603

http://www.morphbank.net/?id=805604

http://www.morphbank.net/?id=805605

http://www.morphbank.net/?id=805606

http://www.morphbank.net/?id=805609

http://www.morphbank.net/?id=805610

http://www.morphbank.net/?id=805612

http://www.morphbank.net/?id=805614

http://www.morphbank.net/?id=805615

http://www.morphbank.net/?id=805616

http://www.morphbank.net/?id=805618

http://www.morphbank.net/?id=805620

http://www.morphbank.net/?id=805621

http://www.morphbank.net/?id=805623

http://www.morphbank.net/?id=805627

http://www.morphbank.net/?id=805628

## Supplementary Material

XML Treatment for Illacme

XML Treatment for Illacme plenipes

## Appendix I

Movie of ♀ *Illacme plenipes* (specimen # SPC000931) with 662 legs showing live movement and head shape. Individual filmed in a glass petri dish with a Nikon Coolpix 995 digital camera mounted to a Leica 12.5 stereomicroscope. (doi: 10.3897/zookeys.241.3831.app1). File format: Apple QuickTime Movie (MOV).

## Appendix II

Movie of ♀ *Illacme plenipes* (specimen # SPC000930) with 666 legs showing very slow, nearly imperceptible locomotion. Individual filmed on an oak leaf with a Nikon Coolpix 995 digital camera. (doi: 10.3897/zookeys.241.3831.app2). File format: Apple QuickTime Movie (MOV).

## Appendix III

Movie of ♀ *Illacme plenipes* (specimen # SPC000930) with 666 legs showing very slow, nearly imperceptible locomotion. Individual filmed on a cardboard sheet with the same method described in Appendix II. (doi: 10.3897/zookeys.241.3831.app3). File format: Apple QuickTime Movie (MOV).

## Appendix IV

Movie of ♀ *Illacme plenipes* (specimen # SPC000931) with 662 legs showing live motion and rapid, independent antennal movement. The species is blind and presumably relies on the antennae to sense its environment. Individual filmed in a glass petri dish with the same method described in Appendix I. (doi: 10.3897/zookeys.241.3831.app4). File format: Apple QuickTime Movie (MOV).

## Appendix V

Times lapse series of visible satellite images of Monterey Bay, California, showing the occurrence of fog extending into the Monterey Basin and Salinas Valley. (doi: 10.3897/zookeys.241.3831.app5). File format: Apple QuickTime Movie (MOV). **Explanation note:** Times lapse series of 330 visible satellite images of Monterey Bay, California, recorded every 15 mins by the GOES-15, Geostationary Operational Environmental Satellite (U.S. National Environmental Satellite, Data, and Information Service) from 10-18 September 2012. Contour lines = 61 m (200 ft). Images provided by the U.S. Naval Research Laboratory, Monterey, California http://www.nrlmry.navy.mil/NEXSAT.html

## Appendix VI

Images of ♀ *Illacme plenipes* (specimen # MIL0020) with 618 legs. Individual photographed with a Nikon D40 dSLR and a 60 mm 1:2.8 AF-S macro lens. (doi: 10.3897/zookeys.241.3831.app6). File format: JPG
