# Supplementary figures and images for: A redescription of the leggiest animal, the millipede Illacme plenipes, with notes on its natural history and biogeography (Diplopoda, Siphonophorida, Siphonorhinidae)
Source: Zookeys. 2012 Nov 14;(241):77–112. doi: 10.3897/zookeys.241.3831 (PMC3559107; doi:10.3897/zookeys.241.3831)

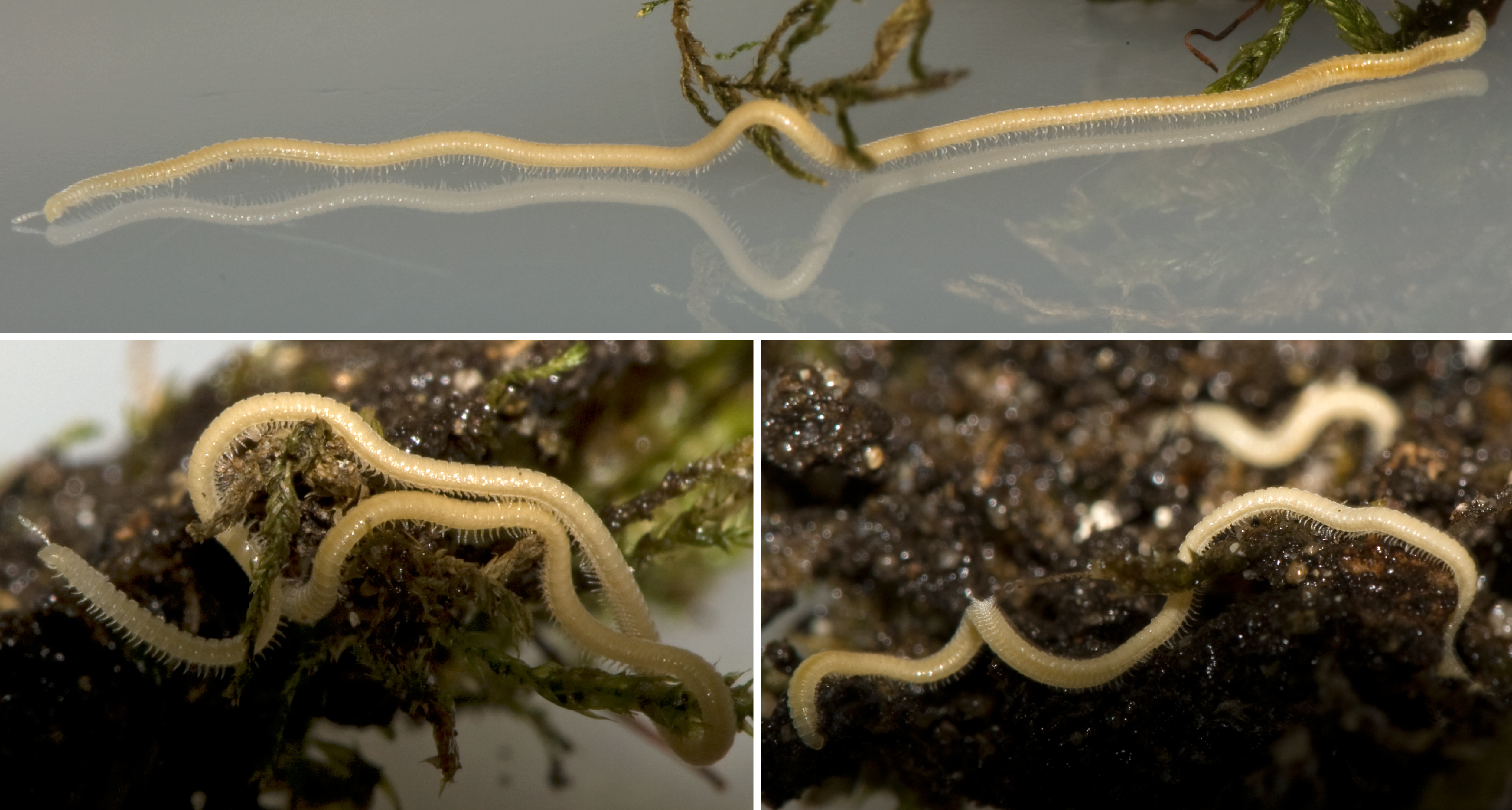

Supplement: Supplementary file 8 — Images of ♀ Illacme plenipes (specimen # MIL0020) with 618 legs. Individual photographed with a Nikon D40 dSLR and a 60 mm 1:2.8 AF-S macro lens. (doi: 10.3897/zookeys.241.3831.app6). File format: JPG [file ZooKeys-241-077-s006.jpg]
